# The Hog Cycle of Law Professors: An Econometric Time Series Analysis of the Entry-Level Job Market in Legal Academia

**DOI:** 10.1371/journal.pone.0159815

**Published:** 2016-07-28

**Authors:** Christoph Engel, Hanjo Hamann

**Affiliations:** Max Planck Institute for Research on Collective Goods, Bonn, Germany; New York University, UNITED STATES

## Abstract

The (German) market for law professors fulfils the conditions for a hog cycle: In the short run, supply cannot be extended or limited; future law professors must be hired soon after they first present themselves, or leave the market; demand is inelastic. Using a comprehensive German dataset, we show that the number of market entries today is negatively correlated with the number of market entries eight years ago. This suggests short-sighted behavior of young scholars at the time when they decide to prepare for the market. Using our statistical model, we make out-of-sample predictions for the German academic market in law until 2020.

## Introduction

What do pigs and law professors have in common? They come in cycles! German academics who observe historical fluctuations on the market for law professors are occasionally overheard mumbling, tongue in cheek: *It’s a hog cycle*. In this paper we read this sigh as a testable proposition. We show that the German market for law professors fulfils the theoretical conditions for the emergence of a hog cycle, and test empirically whether academics allow themselves to be governed by the brute logic of supply and demand, or whether their job market behavior is mitigated by additional forces that are not part of the neoclassical model. This endeavor contributes to a growing literature which addresses the fact that even today we still “know surprisingly little about the market for law professors” [1]:4.

### On pigs…

**Fig 1:**
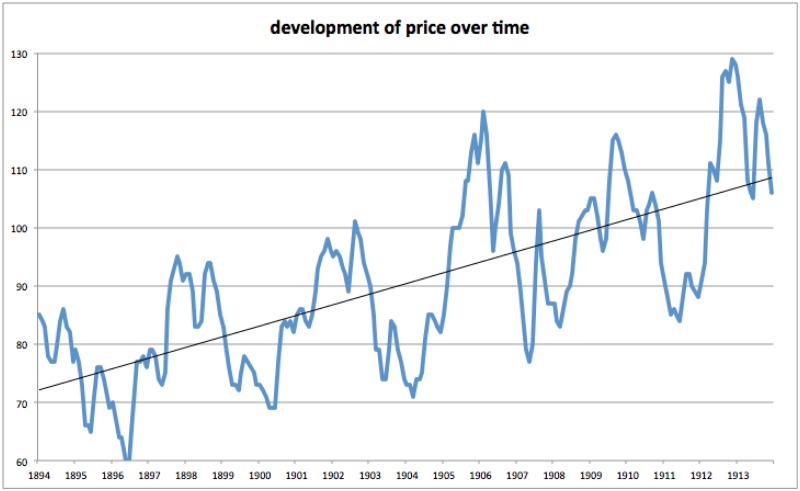
Hog Cycle That Started the Literature ([2]: 10). English translation of German original: Hog Prices in Berlin July 1896 to June 1914 (Pigs with Live Weight 80–100 kg) in Mark [German currency of the time] per 100 kg live weight.

As its name suggests, the concept of “hog cycles” hails from agricultural economics. It describes the idea that once farmers observe a high pork price, they tend to start breeding pigs all at the same time, which means that one year later (when pigs are ready for slaughter) the market will be swamped with pork. Since pork now exceeds demand, its price drops, thus incentivizing farmers to reduce (or cease) their breeding activity, which in turn produces a swing in the opposite direction: A year later, too few pigs are available for slaughter, so demand exceeds supply, prices explode and the cycle restarts. Fig 1 illustrates this principle with the example from early 20^th^ century Berlin (Germany) which ignited academic research on this issue. As one sees, apart from a positive time trend (in the long run, Germans spent more money on buying pork), pork prices were fluctuating cyclically, resulting from cycles of over- and undersupply.

Theoreticians call the underlying logic a cobweb [3, 4]. Three conditions must be fulfilled for a cycle to emerge: (1) in the short run production cannot react to changes in demand; (2) the product in question is perishable; (3) demand in the short run does not increase if there is more supply [3]. Pigs meet all three conditions: (1) Newborn piglets need time to mature; (2) there is the optimal moment for slaughter; (3) if pork is cheap, people hardly eat more. Assume the demand for pork drops unexpectedly, say since consumers dread swine flu. Farmers have to lower prices if they want to sell any pork. Since this meant that profit was low, this year they breed fewer pigs. If these pigs are ready for slaughter, supply is small and prices are high. Farmers react by breeding a larger next generation. When that reaches the market, supply is in excess of demand. What originally was an exogenous shock has triggered a cycle. This holds whenever supply and demand react symmetrically, i.e., the elasticity of demand is similar to the elasticity of supply. If supply is more elastic than demand, the cycle turns into a self-reinforcing spiral. If demand is more elastic than supply, the cycle eventually vanishes [3]. Of course, there is no cycle to start with if a sufficiently large fraction of farmers anticipate this and adapt their breeding policy [5, 6].

These mechanisms are now well understood, thanks to a wealth of academic research. The first papers date back almost a century [2, 7] and were immediately translated into practical advice for farmers, urging them to adopt an anti-cyclical investment policy [8]. Later, Nobel winners Ronald Coase [9, 10] and Paul Samuelson [11] have contributed to this literature, as well as a long list of agricultural economists [12–19]. Similar phenomena have been documented on the market for potatoes [3, 20], for real estate [21, 22], for oil [23], and for nurses [24]. So how about bright young academics?

### … and professors

Given how “little is empirically known about what explains success in the market for law professors” [1]:1, professors-to-be have to take decisions in a complex market based on very few observables. The most salient such observable is the number of positions available when the candidate considers embarking on her academic career. If there are presently more open positions than professors on the market, post-docs may be tempted to start a career in academia. Years later, however, they may find that many others had the same intuition, thereby creating an oversupply which in turn discourages junior researchers and will soon lead to law schools having problems to fill their vacancies. If this were true, we would observe a hog cycle. Of course, future law professors are not hogs, tenure is not slaughter, and hiring a new assistant professor is not breeding. But in theory, the market for law professors fulfils the three conditions, outlined above, which the emergence of a cycle requires.

Note a difference between the proverbial market for hogs, and the market for law professors, though. If there is excess supply, the price of meat may be reduced. If there is excess demand, the price of meat may increase. This channel for short-term adjustment is shut off in the German market for law professors. Prices, i.e. salaries, are defined by statute, and do not react to market fluctuations. In the meat market, the inefficiency results from a mismatch between investment (in rearing piglets) and prices. In the market for professors, the inefficiency results from a mismatch between investment (in habilitation) and the probability of being hired as a professor. Yet as we explain in the following paragraphs, the conditions for a cobweb are no less fulfilled.

(1) Becoming a law professor takes time. In the German system, scholars are eligible only if they have earned their *doctor juris* (Dr. iur., roughly J.S.D. equivalent, not just a J.D.) and have subsequently passed “habilitation” (from Latin *habilitare*, to qualify, [25]). Habilitation is a formal procedure by which the entire professorate of the law school testifies that a candidate is competent to teach law. It requires writing a second book (traditionally called *Habilitationsschrift*, habilitation thesis), having a decent list of publications, and convincing the entire law school during a talk and discussion. Legally, candidates may pass habilitation without having held a post-doc position. The candidate could just submit her thesis, plus her list of publications, to her law school of choice and ask to be habilitated. Yet this legal option is almost never used. One reason is financial: Outside university, there are practically no jobs or stipends for future law professors. The other, more important reason concerns habilitation itself: Getting the whole law school to approve the candidate is a political exercise. The candidate needs strong backing by her supervisor. Effectively, law schools see this as an occasion to express approval or disapproval with the supervisor’s own scholarly work. The candidate is regarded as the supervisor’s pupil, and support for the supervisor’s academic program is at stake. If law schools do not receive this signal of credibility, odds for success are very low. This implies that, when she accepts a candidate as a post-doc, the supervisor engages her own academic reputation. Consequently, preparing for the academic market is practically a joint decision by the supervisor and the candidate.

This explains why the standard career path follows the apprentice system. Some esteemed law professor hires the future colleague as a post-doc (*Habilitand*). Once the post-doc has been hired, it is rare that she leaves academia before finishing habilitation. (There are no statistics on the drop out rate, but our own experience and hearsay suggest that it is negligible). Having spent additional time at the university would not give her an advantage on the market for non-academic lawyers. On the contrary, she would be perceived as less inclined to do practical work, if not poorly equipped for the challenges of legal practice. Holding a post-doc position is thus perceived as a negative signal by the market for non-academic lawyers. Most post-docs would also regard it as personal failure if they did not pass habilitation; they stick to their habilitation plans not least for reasons of self-esteem. The typical post-doc position is for the duration of six years, but finishing the thesis often takes a bit longer (and post-docs and their supervisors creatively need to find her a living for that extra period). According to statistical data reported by Germany’s Federal Bureau of Statistics, the time passed between earning the doctoral degree and passing habilitation was 7.76 years on average over all years for which data is available (1960 to 1989). There is thus a time lag of about eight years between the decision to prepare a new scholar for the market and the moment when she can apply for a position. The short run supply of law professors is inelastic.

(2) By passing habilitation, a young scholar becomes a *Privatdozent*, i.e., an academic entitled to teach, but not usually on a salaried position. Since the law school that granted habilitation is legally prohibited from hiring this candidate itself, the candidate must go on the market and hope to be hired by a different law school. Most *Privatdozenten* are on the market for a year or two. If they have not found a position within three or four years, odds are low that they ever will. The law schools take the fact that a *Privatdozent* has not been hired by other law schools as a signal that something must be wrong with this candidate. Once a *Privatdozent* has taken up a position in legal practice, returning to academia is close to impossible. This makes future law professors a perishable commodity. Note that future law professors also have very little room for changing the moment when they enter the market, in anticipation of changing market conditions. Speeding up the writing process is a frequent plan, but it hardly ever succeeds. Postponing habilitation is usually not possible either, since positions expire.

(3) To date there are only two private law schools in Germany (BLS in Hamburg, EBS in Wiesbaden). The remaining 42 law schools [26] and other departments that occasionally hire lawyers (e.g., business or technology) are funded by the state. This helped law schools during the financial crisis, as their budgets were basically unaffected. Yet it also means that law schools cannot react to an oversupply of excellent candidates by creating new positions. Moreover, professors are public servants, with salaries fixed by statute. Therefore law schools cannot react to excess supply by lowering prices either. Sadly, if there are more good candidates than the market can take, the market does not clear. Those who have not been hired must change their profession. Most of them join law firms. The third condition for a cycle is thus also fulfilled: Demand is not elastic enough to parry fluctuations of supply.

Given that these theoretical conditions are fulfilled, one spark might have been enough to ignite the cycle. In the past, the German market for law professors was hit by exogenous shocks on multiple occasions. The two most prominent shocks happened to be positive ones. In the 1960s and early 70s, the German government decided to invest heavily in university education. No less than 15 new law schools were founded in Augsburg, Bayreuth, Bielefeld, Bochum, Bremen, Düsseldorf, Gießen, Hagen, Hamburg II, Hannover, Konstanz, Mannheim, Osnabrück, Passau and Regensburg. Many additional law professors were needed to fill these new positions. The next shock came with reunification. The former German Democratic Republic had five law schools (Berlin, Halle, Jena, Leipzig and Potsdam), and four more were established shortly after reunification (Dresden, Frankfurt/Oder, Greifswald and Rostock). Since most East German law professors were believed to be too close to the Communist regime, most of them soon lost their positions. They were replaced with candidates from the West. In both periods, demand heavily exceeded supply. In theory, either shock would have been sufficient to start a hog cycle.

In 2002, the position of a “junior professor” was introduced to the German academic system to supplant traditional “habilitation” procedures. Law schools, however, were reluctant to offer such positions: Their number increased from 1 when first introduced to merely 20 at the end of our sample (German Federal Bureau of Statistics schedule H201.250), meaning that no more than 25 lawyers had ever held a junior professor position within our sampling window. Even five years later, in 2014, there were less than 40 positions in total, and virtually none was reported to come with a tenure track option [27]. Passing habilitation remains the only *de facto* possibility to enter the market for professors. Once a scholar has passed habilitation, she is no longer eligible for junior professorship, so these positions cannot absorb excess supply either.

### Hypothesis

Given the theoretical considerations outlined above, we have reason to expect the occurrence of a cycle in the German job market for legal academics, with a duration between six and nine years (eight years being the typical time needed until habilitation). This leads to our hypothesis:

The supply of German law professors exhibits negative autocorrelation with a lag between six and nine years.

To the best of our knowledge, we are the first to put this hypothesis forward, let alone test it. There is one predecessor paper on the German market for law professors, covering the period from 1949 to 1969 [28], but it only reports aggregate descriptive statistics; a more recent empirical study of the US job market for law professors [1] did not gather time series data.

In the remainder of the paper, we describe our data (2.) and explore our hog cycle hypothesis by testing for negative autocorrelation and adding control variables (3.). Finally, we discuss the robustness of our model using additional specifications (4.) and conclude by using our empirical model to predict the supply of law professors for another ten years (5.).

## Materials and Methods

We want to explain the number of habilitations in a given year with the number of habilitations six to nine years prior. We thus need a time series, which we generate from data about individual habilitations.

Ideally we would like to know, for each candidate who has passed habilitation, her name (to double-check with alternative sources), the sub-discipline (private, public, or criminal law), and, most importantly, the year when the candidate passed habilitation. We can get these information from two partly overlapping sources:

A statute from 1969 (see Federal Law Gazette BGBl. vol. I, pp. 265) created the German National Library and obliges all publishing houses to submit at least one copy of each newly published book to that library. Based on these submissions, the “German National Bibliography” has subsequently been compiled and published. If the book in question is a habilitation thesis, this is noted in the bibliography, together with the year of the habilitation procedure. To the extent possible, the statute on the German National Library was applied retroactively. Data for habilitations seem reasonably reliable from 1960 onwards.

Unfortunately, sampling revealed that this data is partly incomplete. To complete the data, we exploit the fact that a Who’s Who of German academics, the *Kürschners Gelehrtenkalender*, was recently made available electronically (www.degruyter.com/view/db/kdgo). In this digest, professors of all disciplines at universities of German language self-report biographical sketches. Since its web interface allowed merely the exporting of names, not the year of habilitation, the sub-discipline and the university, we had to complete these data points by hand. Hand coding was also necessary to identify persons who had been invited to contribute to the digest without having passed habilitation. This mainly concerns honorary professors, i.e., practitioners who receive the honorary title of a professor in exchange for teaching students free of charge. We have removed such entries from our dataset.

We have matched these two datasets by name and removed all duplicates. The resulting gross dataset contains 2071 data points. We thus have complete data of 2071 habilitations for the time between 1876 and 2009. (Data from 2010 onwards is still too incomplete to include.) However, we truncate our dataset in 1960, before which year we cannot rely on the records to be complete. This leaves us with 1993 data points, which are available (with control variables included) as S1 File. There is a predecessor publication reporting descriptive statistics of habilitations in law from World War II until 1969 [28]. It relies on a survey, but unfortunately reports only aggregates. We therefore cannot use this publication to extend our time series further into the past.

In our regressions, we work with the total number of habilitations per year. Our original dataset comprises 50 observations. Depending on the length of the lag, it is reduced to the number of years for which we can observe the lag. Given the inevitably small number of observations, it is all the more remarkable that we find very robust results.

Finally, to identify sub-disciplines, we have double-checked our data with lists compiled by the associations of private-law professors (website of *Zivilrechtslehrervereinigung*, www.zlv-info.de, accessed on 1 Oct 2010), of criminal-law professors (courtesy of Prof. Bernd Schünemann in private correspondence on 20 Jan 2011), and of public-law professors (website of *Vereinigung der Deutschen Staatsrechtslehrer*, http://vdstrl.zar-muenster.de, accessed on 1 Oct 2010).

**Fig 2:**
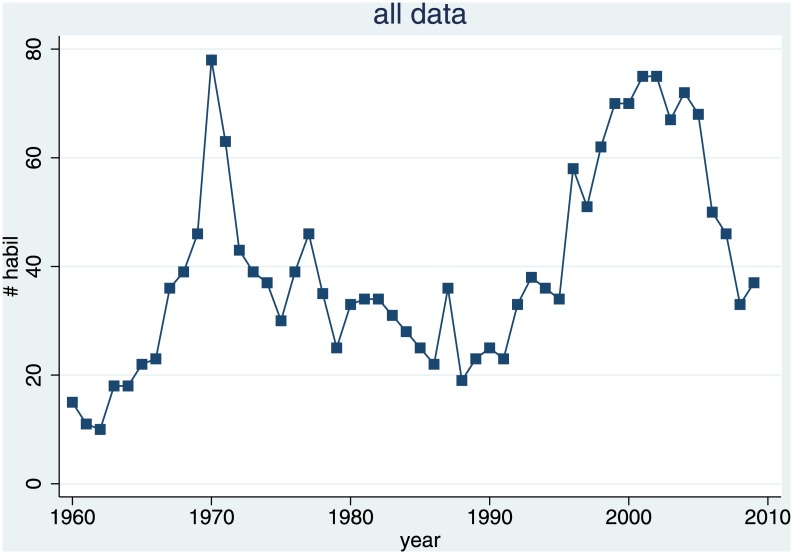
Production of Law Professors. Total number of habilitations per year.

Fig 2 shows that the production of law professors has not been smooth. There was a first spike in the early 70s and a second shortly after the year 2000. In the intermediate period, and possibly also in the current years, there is a much smaller supply of law professors. Note that visual inspection of the raw data already suggests a similarity with the hog cycle graph in Fig 1.

**Fig 3:**
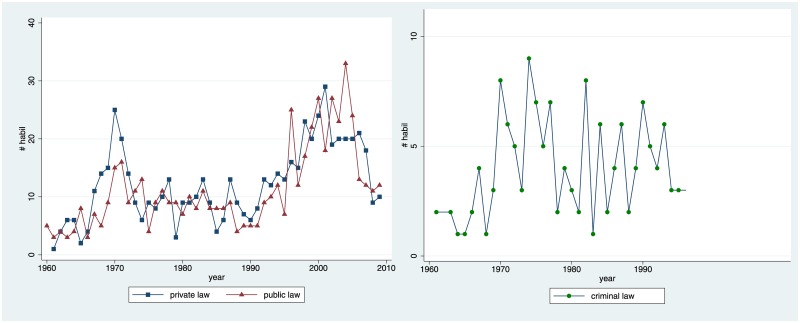
Development in the Three Institutionalised Sub-disciplines. Total number of habilitations per year.

Fig 3 demonstrates that the development in the sub-disciplines was very similar. The only remarkable difference concerns the early spike (which was more pronounced in private law) and the recent spike (which was more pronounced in public law). Furthermore, habilitation data in criminal law is noisier, which may be attributed to the comparatively small number of law professors organized in this association. The overall shape of the curve is nonetheless similar. Since sub-disciplines do not make a pronounced difference, in the remainder of the paper we work with the complete dataset.

## Results

### Autocorrelation

If there is a hog cycle of law professors, the supply of professors today must be negatively correlated with the supply of professors x years ago. As a first test for our hypothesis, we estimate time series regressions with an autocorrelation term. Specifically we estimate

$$y_{t}=\alpha +\rho y_{t-x}+\varepsilon_{t}$$ (1)

where *y*_*t*_ is the number of habilitations in year *t*, *α* is a constant term, *y*_*t-x*_ is the number of habilitations *x* years ago, and *ε*_*t*_ is residual error. The coefficient of interest is *ρ*, which we expect to be negative and significant. The peaks in Fig 2 suggest that the data might be heteroskedastic, which is why we estimate Huber-White robust standard errors.

**Fig 4:**
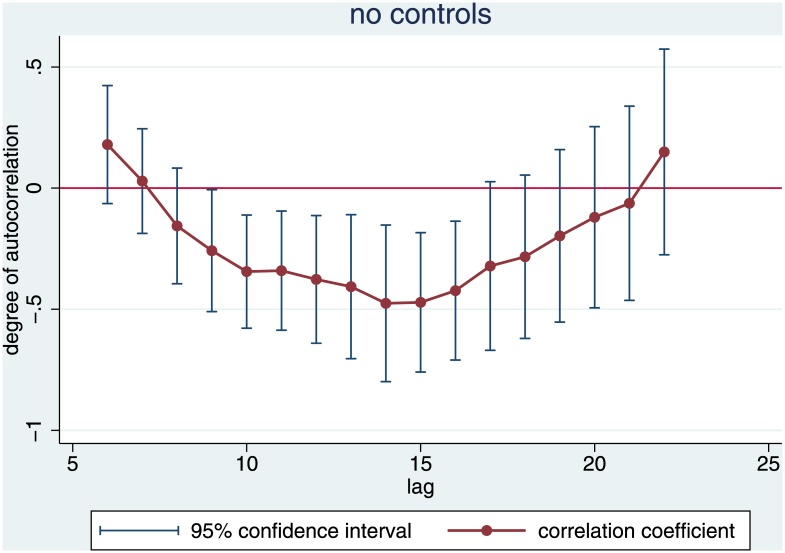
Autocorrelation (absent controls). Dots are coefficients of the lag of the number of habilitations x years ago from regression (1), bars represent the standard error of this regressor.

Autocorrelation requires the coefficient for the number of habilitations x years ago to be significantly different from zero. Fig 4 compresses the findings from 17 time series regressions with different lags. It illustrates two findings: First, all coefficients for lags between length 8 and 21 are negative, i.e., below the zero line. We thus do find a hog cycle. If there have been many habilitations in the past, the statistical model predicts few habilitations today, and vice versa. This is what we expected. However, the error bars indicate that only lags from nine to 16 years are significantly different from zero.

For the lag at the upper end of our expectation, i.e., a lag of nine years, the autocorrelation is weakly significant (p = .051), see Table 1. This model predicts that there are 52.82 new law professors on the market every year, minus .258 times the number of habilitations nine years ago. Since this number has never been zero, the predicted number of habilitations is always smaller than 52.82 –but the more so, the larger the supply in the past. The negative correlation is pronounced. Four habilitations in the past seem to deter one habilitation now.

**Table 1:** Pure Autocorrelation.

lag9 habil	-.258^+^
cons	52.816***
N	41
p model	.051
R^2^	.0585

depvar: # habilitations in year x, robust standard errors

*** p < .001,

** p < .01,

* p < .05,

^+^ p < .1

Our data thus support the theoretical prediction of negative autocorrelation, for a lag at the upper end of our expectation. Yet deeper lags are also significant, and there are also statistical reasons to revisit the estimation. Even the best-performing model with one lag explains only 13% of the variance. Obviously a lot of the fluctuation in the supply of law professors is still unexplained. More importantly even, as long as we miss those additional factors that determine the development of habilitations over time, the coefficients of that time lag(s) in Table 1 risk being inconsistent due to omitted variable bias. In the next section, we introduce control variables to mitigate this bias.

### Control Variables

**Fig 5:**
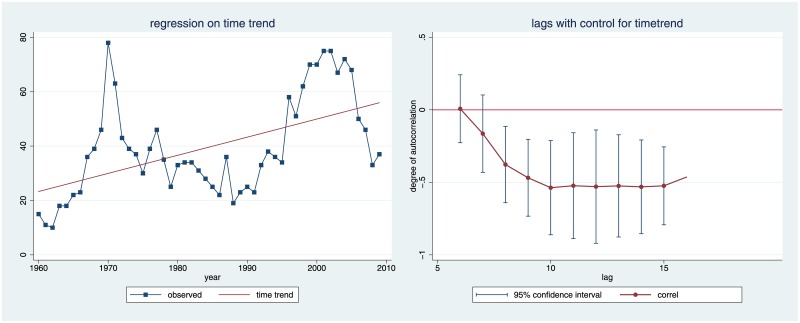
Time Trend.

Fig 5 shows that, despite the fluctuations, the number of habilitations grows over time. (The red line graphs the predicted number of habilitations if we regress the output of law faculties only on the respective year.) Over the 50 years of observation, legal academia has been growing.

**Fig 6:**
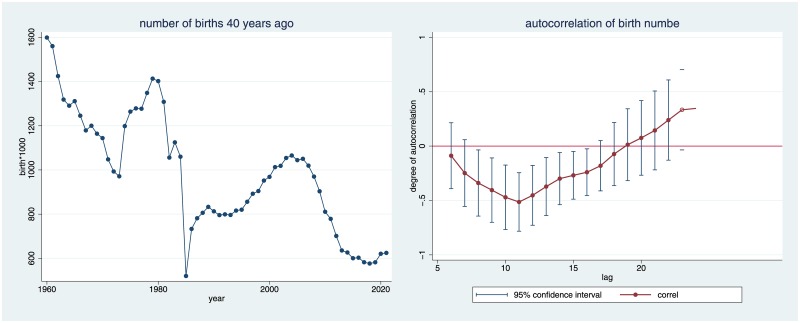
Development of Birth Cohorts over Time.

The German apprenticeship model of academic careers takes considerable time. Data by the German Federal Bureau of Statistics (schedule 4.15.2a) show that between 1980 and 2008, the mean age at habilitation was 39.76 (standard deviation .92), so future law professors are around the age of 40 upon entering the market. One should expect that there are more candidates the larger the respective birth cohort is. The number of those who bring the right talents should be more or less proportional to the number of those born forty years before. Consider Fig 6, which depicts data on birth cohorts from the German Federal Bureau of Statistics.

Fig 6 illustrates why birth cohorts are relevant for understanding the production of law professors. The data contain several structural breaks: one drop in birth cohorts, presumably caused by the economic crisis in the early 30s (playing itself out in the early 70s), and an even more pronounced dip, which resulted from the end of World War II (and played itself out in the mid-1980s). Conversely, steep increases resulted from the baby booms in the early Nazi years (becoming effective in the late 70s) and during the recovery after World War II (peaking in the early 2000s). Overall, there is the clear negative trend characteristic of modern affluent societies.

The right panel of Fig 6 is even more important. It shows that birth numbers also exhibit autocorrelation. The autocorrelation is negative in the short run, and most pronounced around eleven years, and positive in the long run, with a peak at 27 years. Now the original time series regressions from Fig 4 cannot discriminate between the two channels of negative autocorrelation: the one resulting from parents’ decisions on family planning, and the other resulting from the future candidate’s decisions on her own career. Controlling for the size of birth cohorts will isolate the latter effect.

The supply of professors need not be proportional to the size of the birth cohort. It would only be proportional if the composition of professions in the population were stable over time. Now, in the period of observation, Germany has experienced what is often referred to as the “education revolution”. While at the beginning of our time series only little more than 5% of a cohort passed high-school exams (*Abitur*) and were therefore eligible for university education, this number grew steadily and has reached almost 25% today [29]. If the supply of professors is proportional to those members of a birth cohort who go to university, rather than to the total size of the cohort, we still have an omitted variable.

We therefore use the size of the student cohort of which the future law professor is part as an additional control variable. Specifically, we use the number of students who were enrolled in German universities 20 years ago. We take this data from the German Federal Bureau of Statistics. The lag of 20 years is motivated by the fact that a typical law professor is around 40 when she is on the market, and that Germans typically enter university around age 20.

**Fig 7:**
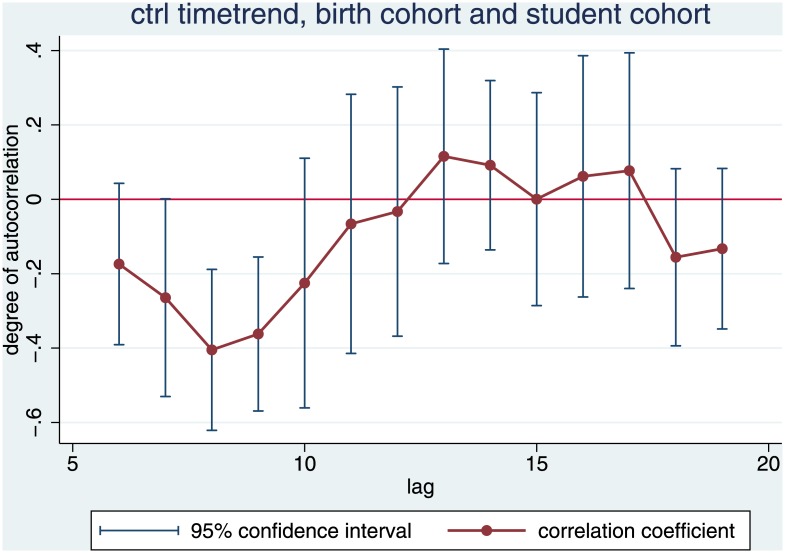
Adding Time Trend, Birth Cohort and Student Cohort. Dots are the coefficients of the lag of the number of habilitations x years ago, bars represent the standard error of this regressor.

If we add the time trend, the birth cohort 40 years ago, and the student cohort 20 years ago to the specification of (1), only shorter lags of the number of habilitations turn out significant. The best performing model now has a highly plausible lag of eight years (Fig 7).

The regressions in Table 2 show that, once we control for the size of the student cohort, the effect of the birth cohort becomes immaterial. In model 2, the regressor of the birth cohort is insignificant. Adding this control variable does not explain any additional variance. Two more findings are remarkable. With these regressions, we explain a huge proportion of the variance, namely more than 75%. Moreover, once we control for the size of the student cohort, the overall time trend becomes negative. Student cohorts grow faster than calendar time.

**Table 2:** Adding Time Trend and Student Cohort.

	model 1	model 2	model 3
depvar	# habil	# habil	student cohort
time trend	-2.772***	-2.366***	2.210***
student cohort lag20	1.659***	1.522***	
birth cohort lag40		.011	.021***
lag8 habil	-.335***	-.405**	
cons	68.087***	53.138**	-44.277***
N	42	42	42
p model	< .001	< .001	< .001
R^2^	.7738	.7808	.9353

depvar models 1 and 2: # habilitations in year x, robust standard errors; depvar model 3: student cohort, 20 years ago; time trend is 0 in 1960, and grows by 1 every year; student cohort is # of newly inscribed students in all German universities, 20 years ago; birth cohort is # of births (in 1,000), 40 years ago

*** p < .001,

** p < .01,

* p < .05

Actually we can even be more sophisticated. In model 3 of Table 2, we regress student cohorts 20 years ago on birth cohorts 40 years ago and the time trend. Both coefficients are significant, and have the expected signs: student cohorts grow over time, and they grow faster than birth cohorts (which is why, even when controlling for birth cohorts, the time trend remains significant and negative). In the last step, using the procedure introduced by [30], we can test whether birth cohorts have a significant indirect effect on the number of habilitations, mediated by student cohorts (for background see [31]). This turns out to be the case (z of indirect effect 3.867, p = .00011). Thus birth cohorts matter chiefly because they affect how many students go to university, which in turn affects how many graduates can become professors.

### Alternative Career Options

Arguably, when they consider preparing themselves for a career in academia, promising young lawyers consider attractive alternative career options. Recall that, once they have become a post-doc, it is very unusual for lawyers to leave university and go to practice (unless forced to do so since, post-habilitation, they cannot find a job at a university). Therefore the relevant moment for exploring alternative career paths is after having earned their *Dr*. *juris*, and before taking up a post-doc position.

In the German system, future law professors typically consider one of two alternative career paths. Those who are interested in earning money would join one of the big law firms. Those more interested in advancing justice, serving their country, or maybe having a couple of children soon (note that the system of maternity leave is most generous for public servants), would rather join the judiciary. In Germany, lawyers may become judges right after having finished their legal education. After the age of 32, the judiciary normally no longer hires, meaning that lawyers stand little chance of becoming judges should they pass habilitation, but are not hired by a law school. All over the country, there are more than 20,000 judges. Careers in the judiciary tend to be slow, but can ultimately lead to the Supreme Court or to one of the prestigious appellate courts. Salaries are rather modest and fixed by statute. Given that we have already seen that a lag of eight years is most relevant, we also check for career opportunities eight years before a generation of candidates is on the market.

**Fig 8:**
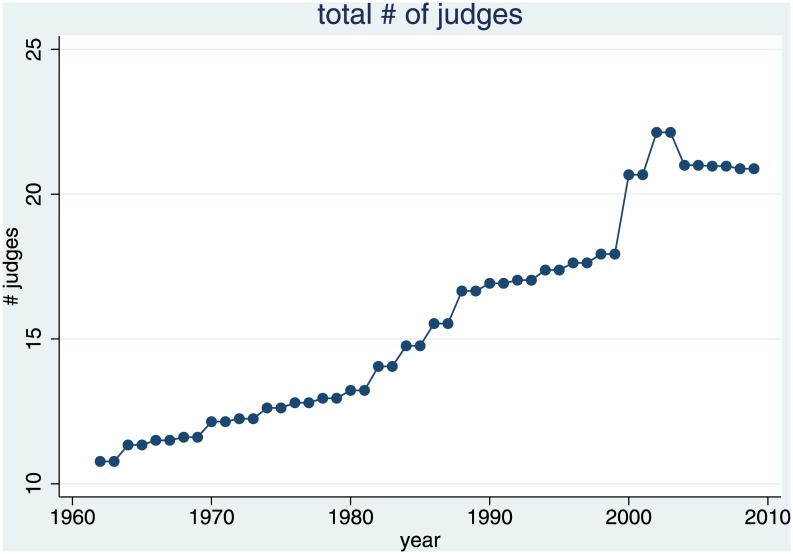
Number of Judges in Germany (in 1,000).

Entry into the judiciary critically depends on hiring. As Fig 8 shows, until the mid-90s, the number of judges grew slowly, with the only peak resulting from reunification. We take this data from the biennial employment reports of the German Federal Ministry of Justice.

**Fig 9:**
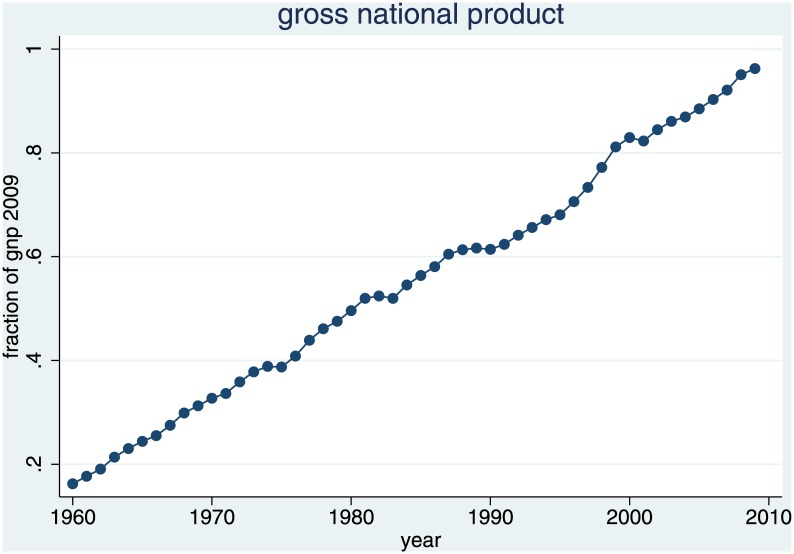
GNP Development as a Proxy for earning Prospects in Law Firms.

As a proxy for earning prospects in the big law firms, we take the development of gross national product; unfortunately, no major law firm has been both willing and able to give us time series data on salaries for associates, or on the number of associates hired per year. As Fig 9 shows, GNP has been growing almost linearly year by year. The turnover of law firms should be positively correlated with the ability of potential clients to pay higher fees, which in turn should be correlated with GNP.

The regressions in Table 3 make it clear that alternative career options are not an important determinant in the decision to prepare for an academic career in law. If we do not control for the size of the respective student cohort, in models 1 and 3, we do find a significant effect of these regressors, but it has an unanticipated sign, suggesting that alternative career options make rather than break the case for academia: If the judiciary is large at the moment when a young lawyer decides about her career, she is *more* likely to become a professor. Likewise, the larger the gross national product is at this point in time, the *more* she is likely to forego the earning opportunities in the law firms, and to start an academic career instead. The effect of the total number of judges disappears once we control for the size of the student cohort (model 2). The effect of GNP goes down to about a third of its previous size, but remains significant (models 4). The effect of the number of judges in model 1 partly picks up the effect of student cohorts, suggesting a correlation about the causes of which we could only speculate. The higher the GNP is, the more habilitations occur eight years later, as shown in model 3. The remaining effect of GNP in model 4 could reflect the expectation of future law professors that a more prosperous country will continue the expansion of the university system.

**Table 3:** Adding Measures for Alternative Career Options.

	model 1	model 2	model 3	model 4
time trend	-1.820*	-2.469***	-6.361***	-5.029***
# judges	9.026**	-1.901		
GNP			447.808***	162.010*
student cohort lag20		1.767***		1.520***
lag8 habil	-.258^+^	-.358***	-.557**	-.404***
cons	-41.831^+^	86.836***	-31.221*	40.544**
N	42	42	42	42
p model	< .001	< .001	< .001	< .001
R^2^	.3645	.7782	.3997	.7944

depvar: # habilitations in year x, robust standard errors; time trend is 0 in 1960, and grows by 1 every year; student cohort is # of newly inscribed students in all German universities, 20 years ago; # judges eight years ago; GNP eight years ago, in % of GNP 2009

*** p < .001,

** p < .01,

* p < .05,

^+^ p < .1

### Available Positions in Academia

When professors offer a promising student a post-doc position, and when this student decides to accept the position and to prepare for an academic career, it would be rational for both to consider the number of positions that will be available when the candidate is on the market, i.e., some eight years from then. If this number is constant, this is of course not a concern. But the two shocks that drive the peaks in Fig 2 result from the rapid expansion of public universities. In the 1970s, policy makers wanted to educate a much larger fraction of the population. In the 1990s, reunification led to a high demand for new professors. In response to both shocks, individuals from some birth cohorts were much more likely to become professors than academics who were a few years older or younger. In German public universities, the mandatory retirement age is, once again, 68 (with a sustained interlude where retirement age has been lowered to 65). Due to this, the demand for law professors predictably peaks when one of the cohorts hired during one of the two shocks is up for retirement.

**Fig 10:**
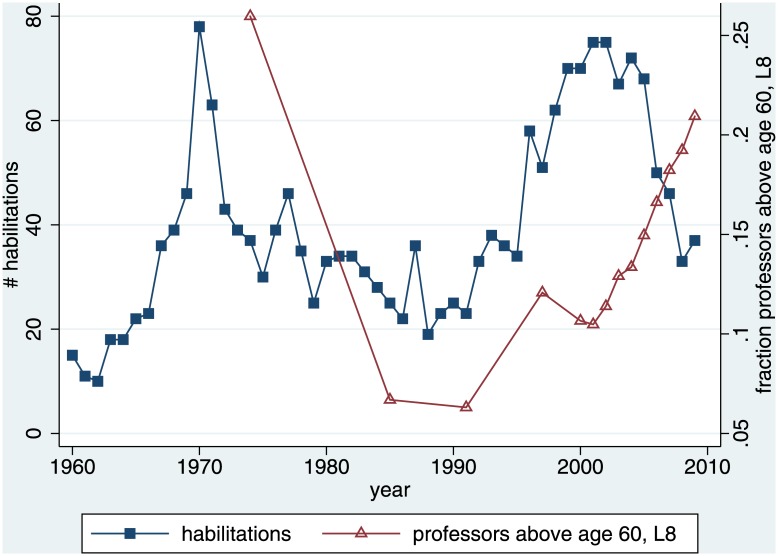
Expected Number of Available Positions.

Data about open positions is only available from 1992 on. Since we need an eight-year lag, this would reduce our time series to only nine years. We therefore work with the share of professors aged 60 and above. This data has been reported by the German Federal Bureau of Statistics since 1966 (series 11 schedule S.1 prior to 1990 and schedule 4.4 subsequently), although initially only for selected years. Descriptively it seems as if this information might have had some influence after the first peak. By contrast, the second peak seems to have been unaffected (Fig 10).

Yet model 2 of Table 4 suggests that the number of habilitations is not driven by the anticipated development of open positions eight years ahead. The regressor is insignificant. Adding this control variable explains less than an additional percent of the variance, in a model that already explains 88% (model 1). The coefficients of the remaining control variables change very little. Most importantly, the eight-year lag of the dependent variable becomes even more pronounced. We can therefore exclude that the hog cycle is spurious. The impression of a cycle does not result from the fact that those considering preparing for the academic market do something very sensible, namely react to predicted volatility of demand.

**Table 4:** Adding a Proxy for Available Positions in Academia.

	model 1	model 2	model 3
time trend	-4.369***	-4.615***	-4.651***
student cohort lag 20	2.190***	2.195***	2.076***
fraction professors above 60 lag 8		82.481	
absolute number professor above 60 lag 8			.016
lag8 habil	-.370**	-.462**	-.655*
cons	98.164***	101.176***	117.940***
N	28	28	28
p model	< .001	< .001	< .001
R^2^	.8810	.8881	.8913

depvar: # habilitations in year x, robust standard errors; time trend is 0 in 1960, and grows by 1 every year; student cohort is # of newly inscribed students in all German universities, 20 years ago; model 1 repeats model 1 of Table 2, but cuts the time series to the 28 data points for which we have the additional control variable that we use in model 2; fraction/number of professors 8 years ago: missing data replaced by linear imputation, using last reported value + ((next reported value–last reported value)/(number of missing values))*(position in this series of missing values)

*** p < .001,

** p < .01,

* p < .05,

^+^ p < .1

The decision to embark on an academic career might also be influenced by the absolute number of professors close to retirement, rather than their share. This would imply that the future professors are less sensitive to the overall turnaround, and would rather care about how many opportunities they expect to exist for applying. Model 3 of Table 4 demonstrates that this information does not have a significant effect either. In this specification, the eight-year lag of the dependent variable becomes even more pronounced.

## Model Selection

The previous analysis indicates that a model controlling for the time trend and the student cohort twenty years ago, plus an autocorrelation term with a lag of eight years, best explains our data. This fully supports our hypothesis. Since the lag is highly significant and substantially negative in this model, this suggests that there is indeed a hog cycle, triggered by perceived job opportunities at the moment when the future professor and her supervisor decide to have her prepare for entering the market. We now undertake a number of robustness checks to corroborate our result.

As we have discussed near the end of Section 2 and illustrated in Fig 2, our dependent variable reacted positively to two strong exogenous shocks in the early 1970s and 2000s. If our hypothesis holds true, promising young lawyers should not only be influenced in their decision to prepare for an academic career if they observe such salient shocks. As a first robustness check, we thus control for the effect these two shocks have had *per se* (apart from their ability to trigger a cycle). Model 3 below enriches our model with dummy variables that neutralise the two peaks exhibited by our data in Fig 2. (Note that the second peak stretches over more than one year. Our dummy flags the first year in which the number of habilitations was close to maximum, but results do not change for any other year during that peak.) These additional control variables are indeed significant, but they do not affect our variable of interest, the eight-year lag.

**Fig 11:**
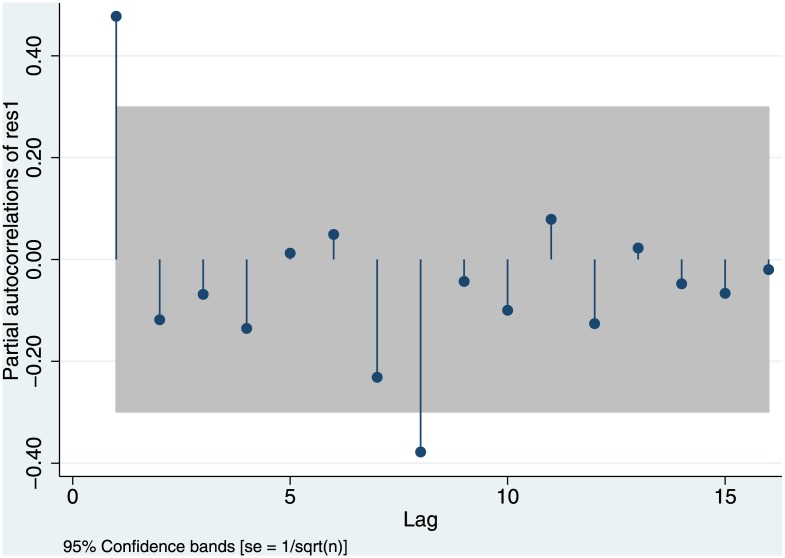
Partial Autocorrelation of Residuals. From OLS of # of habilitations in year t, controlling for # of new students in t-20 and the time trend.

As another check of robustness, we examine a partial autocorrelation plot. Since we already know that there is a time trend and that we must control for the size of student cohorts, we do not base this plot on the raw data, but on the residuals from a regression that controls for these two variables (but of course not for the eight-year lag). Partial correlations measure the correlation of the number of habilitations in any given year with the number of habilitations n years ago, after the effect of all shorter lags has been partialled out. As one sees, the negative correlation with the number of habilitations eight years ago indeed stands out (see Fig 11). This demonstrates that we are justified in using the eight-year lag, rather than lags of alternative duration.

Fig 11 suggests that, additionally, there is positive autocorrelation with the year before. This is plausible. Habilitation procedure is complicated. Candidates starting in the same year may not all be able to finish their habilitation thesis at the same moment, and some law schools are better than others at organizing the formal procedure. As a first robustness check, we therefore estimate a model with an additional autocorrelation term for the one-year lag (Table 5, model 2). The additional lag is indeed significant, but adding this additional control variable does not affect the significance or the size of the negative eight-year lag.

**Table 5:** Alternative Specifications of Preferred Model.

	model 1	model 2	model 3	model 4	model 5	model 6
depvar	# habil	# habil	# habil	# habil	# habil	# habil
time trend	-2.772***	-1.740*	-1.252**		-2.434***	-3.044***
student cohort	1.659***	1.066**	.829**	.194**	1.468***	1.744***
lag1habil		.352*	.421**	-.346***		
lag8habil	-.335***	-.303**	-.244**	-.238**	-.594***	
year1970			27.089***	32.770***	21.249*	17.420*
year1998			7.783**	7.715**	1.391	7.082
ma(1)					.380	
lag8habil, instrumented by lag10habil						-.293**
cons	68.087***	46.003**	35.648***	14.993**	53.790***	71.142***
N	42	42	42	42	42	40
p model	< .001	< .001	< .001	< .001	< .001	< .001
R^2^	.7738	.8095	.8636	.5459		.8479

depvar: # habilitations in year x, robust standard errors; time trend is 0 in 1960, and grows by 1 every year; student cohort is # of newly inscribed students in all German universities, 20 years ago; year1970 is an indicator variable that is 1 for habilitations in 1970; year1998 is an indicator variable that is 1 for habilitations in 1998; ma(1) is a moving average term, of length 1 year

*** p < .001,

** p < .01,

* p < .05

In our preferred model, we use the time trend for detrending the data, since this gives coefficients a straightforward interpretation. In the time series literature, detrending is typically done through first differencing. If we use this procedure, we again support the significant negative eight-year lag, model 4.

A model with an autocorrelation term assumes that the effect of a past shock only gradually fades away. Our explanation for the significance of the one-year lag would also fit a model with a moving average term, which is assumed to become immaterial after the period affected is over. Model 5 shows that the moving average term is indeed positive and significant but that, in this model, the p-value for the eight-year lag is even smaller. Since we want to jointly estimate a moving average term and an autocorrelation term, in this model we represent the autocorrelation by the lag in residuals, not in the dependent variable (i.e., we use Stata command *arima*), and find that deeper moving average terms are insignificant.

Finally, in model 6 we instrument the eight-year lag by the ten-year lag that our earlier results show to be clearly uncorrelated with the dependent variable, i.e., with the number of habilitations today (after controlling for the time trend and the size of the relevant student cohort). This way we guard against the possibility that omitted variables make our estimation of the eight-year lag inconsistent. Although in this model we only have the correlation between our instrument and the eight-year lag for explanation, the effect of the eight-year lag still is highly significant–and remains significant if we add the 11-year lag as an additional instrument. Note that this procedure is in the spirit of the Arellano Bond model. The Arellano Bond model is concerned with possible inconsistency resulting from patterned heterogeneity between individuals (cross sections) in a panel. Heterogeneity can be interpreted as an omitted variable. Instrumenting with lags of the dependent variable one has reason to believe to be uncorrelated with contemporaneous choices removes the resulting risk of inconsistency. We do not have a panel, so that inconsistency cannot result from unobserved heterogeneity. But past lags as internal instruments are also valid in a mere time series model. Yet, since we need not remove individual specific effects, we have no reason to first first-difference the data.

Summing these additional tests up, we find that our main result, the eight-year lag, is very robust to changes in the specification of the statistical model. Note that all robustness checks leave the significant eight-year lag of the independent variable unaffected, even if we perform them on model 2 of Table 4. To avoid working with this considerably shorter time series (and partly imputed data for the percentage of professors above 60) we have presented robustness checks only on the model without this additional control variable, but the alternative robustness checks are available from the authors upon request.

## Conclusions

Currently the entry-level job market in legal academia “remains largely a black box” for which “we do not know the factors that influence how law schools and candidates make decisions” [1]:5. To contemplate some such factors and to lift the lid on the black box of these job market decisions, we have shown that the market for law professors fulfils the conditions for a cobweb, namely inelasticity of supply and demand as well as perishability: Preparation for an academic career in law takes many years. Therefore supply cannot swiftly adapt to changes in demand. The (German) law faculties are hesitant to hire candidates who have been on the market for more than a small number of years. The faculties tend to read this as a signal that something must be wrong with this candidate. Therefore future law professors are a perishable commodity. Finally, the (German) law faculties live off public funds and must pay salaries that are fixed by statute. The faculties are therefore unable to respond to excess supply by hiring more professors. Theory thus predicts a hog cycle: When there have been too few law professors in the past, there is too much supply today. When there have been too many law professors in the past, there is too little supply today.

**Fig 12:**
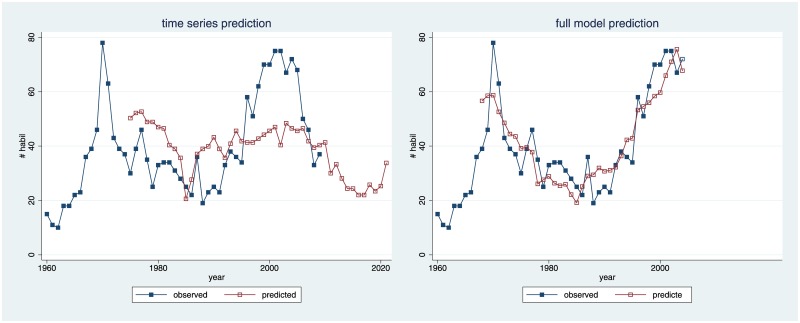
Out-of-sample Predictions. (A) Left panel is based on the best-performing model of Fig 4, i.e., on mere autocorrelation with a 15-year lag. (B) Right panel is based on final model, i.e., on model 1 of Table 5.

In this paper, we exploit the fact that, in the German university system, future law professors must formally qualify for the academic market by passing habilitation. The decision to grant habilitation is taken by the law school of origin, which is prevented from hiring its own candidates. We are thus in a position to quantify precisely the supply of candidates for all the years since 1960. We find significant autocorrelation, but time lags alone explain little of the variance in our data. This changes if we control for the overall time trend and for the size of student cohorts. Fig 12 summarizes our results. A mere time series model (left panel) has a relatively poor fit, despite the fact that the lag of 15 years is highly significant. By contrast, the model controlling for the overall time trend and the size of student cohorts, with autocorrelation of eight years (right panel), has an excellent visual fit.

In our final model (see Table 5, model 1), we find significant negative correlation between the number of habilitations eight years ago and today. The correlation coefficient is -.335, i.e., three more habilitations eight years ago approximately deter one habilitation today, and three fewer habilitations eight years ago encourage one more habilitation today. This suggests that, when a supervisor approaches a promising candidate, and when this candidate accepts the offered post-doc position, both are overly sensitive to current experiences on the academic market. If law schools currently have a hard time filling open positions, and if those who have just passed habilitation quickly find a good job, supervisors and candidates infer that there is demand for more law professors. By contrast, if one open position currently draws 50 or 60 applications, out of which many are serious candidates, supervisors are hesitant to invite promising young lawyers to embark on such a hazardous career, or candidates turn down offers for that reason.

Fig 12 also serves another purpose. We can use either model to make out-of-sample predictions for 10 years after the end of our time-series. Given our findings, one should not trust predictions from the mere time series, but should also take student cohorts into account, and should control for the time trend. Student cohorts of those researchers applying for open positions at the law faculties between 2010 and 2020 are already known. Using this information, our model predicts another steep increase until 2018, with some 85 candidates.

Shall we use this information to repeat the advice Fritz Baade and Hermann Abeking gave hog farmers in the 30s of the previous century [8]? They urged them to adopt an anti-cyclical investment policy. In our case, this would mean that promising young lawyers should be hesitant to embark on an academic career (and supervisors should hesitate to encourage them) when present job opportunities are plentiful. If our regression has it right, when these lawyers are ready for the market of professors, good candidates will be in excess supply. There are two reasons why such conclusions ought to be treated with more caution, though. It is not clear how fast and how profoundly the demand side of this market is changing. Finance ministers may be forced, after the financial crisis, to cut back on all public expenses, university education included. Law faculties may be forced to react to severe cuts by structural reforms. Such reforms are likely to privilege cheaper new candidates over the promotion of more established professors.

Moreover, an anti-cyclical reaction is only individually beneficial if most others behave cyclically. Theoretically, the resulting problem is well understood. It is known in game theory as the beauty contest, following John Maynard Keynes’ famous likening of the stock market “to those newspaper competitions in which the competitors have to pick out the six prettiest faces from a hundred photographs, the prize being awarded to the competitor whose choice most nearly corresponds to the average preferences of the competitors as a whole” [32]. Keynes anticipated what later got generalized as the theory of level-k-reasoning [33, 34]. It considers self-referential decision processes, where decision outcomes depend on the expected decisions of others in the same situation, and assumes that players determine best replies to each other’s behaviour by reasoning iteratively. For instance, let a simpleton with no regard for fellow players’ strategies be defined as a level 0 player, then any player who anticipates and reacts to level-0-reasoning would inhabit level 1. Even more sophisticated strategists would ascend to level 2 by anticipating level-1-reasoning, and so on. In its general form, any level-k-strategy assumes that all other players use reasoning levels between 0 and (k-1). Empirical research suggests that most people only use a degenerate version of this iterated best reply mechanism, with level-1 and level-2 being most prominent (for a comprehensive survey, see [35]).

While this literature suggests that the foresight of most people is rather limited, is it plausible to assume that the large majority of future law professors, and of their supervisors, is not clear-sighted at all? Probably not, as the studies mentioned above even found a remarkable proportion of people adopting level-∞-reasoning, depending on their respective training, time availability, effort, and judgment confidence. On the other hand, for future law professors the problem is exacerbated by the fact that they must predict a market some eight years ahead. Therefore the strategic uncertainty inherent in the beauty contest is compounded by the stochastic uncertainty inherent in all sorts of changes that may happen in the meantime.

In principle, government could step in and limit the number of positions available for future candidates for habilitation. This number could correspond to the expected number of open positions eight years ahead. Yet for a number of reasons, this solution would be problematic. In many domains, government forecasts of future developments have widely gone wrong. If the number of positions available for habilitation is determined by law, there will be strong pressure for candidates indeed to be hired once they have passed habilitation. This would cut into the budget autonomy of the legislator. Most importantly, this solution would profoundly change universities. One would need a nationwide competition for the positions that are made available. Effectively, the decision who is going to be a professor would be taken after this individual has acquired her doctorate, not after habilitation. Essentially, Germany would have to shift to the tenure-track system. There is debate whether this system is preferable. But breaking the hog cycle of candidates is certainly not a sufficient reason for making this move.

We must therefore leave young lawyers considering an academic career with a problem of judgement: If they want to rely on our findings, they must also estimate how many of their potential competitors will try to second-guess the market at the moment when they expect to enter it. Ultimately, the traditional piece of advice will remain best: Only prepare for an academic career in law if your advisor truly believes in you, and if you prefer a life driven by academic curiosity over the alternative options for a good lawyer.

## Supporting Information

S1 File Comprehensive dataset in Stata.dta file format. (DTA)

S2 File Data analysis algorithms in Stata.do file format. (DO)
