# Correction: The Hog Cycle of Law Professors: An Econometric Time Series Analysis of the Entry-Level Job Market in Legal Academia

**DOI:** 10.1371/journal.pone.0168041

**Published:** 2016-12-09

**Authors:** The PLOS ONE Staff

There are formatting errors in Figs 3, 5 and 6. Please view the corrected figures here. The publisher apologizes for the error.

**Fig 3:**
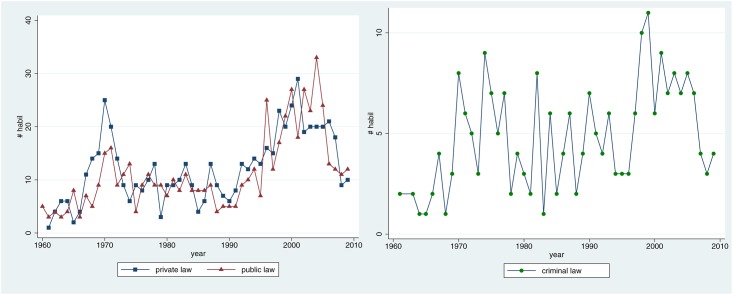
Development in the three institutionalised sub-disciplines. Total number of habilitations per year.

**Fig 5:**
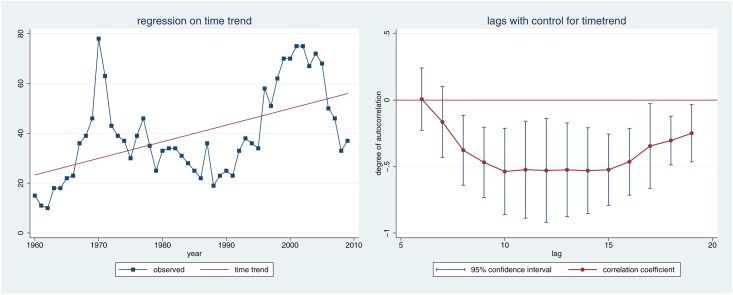
Time trend.

**Fig 6:**
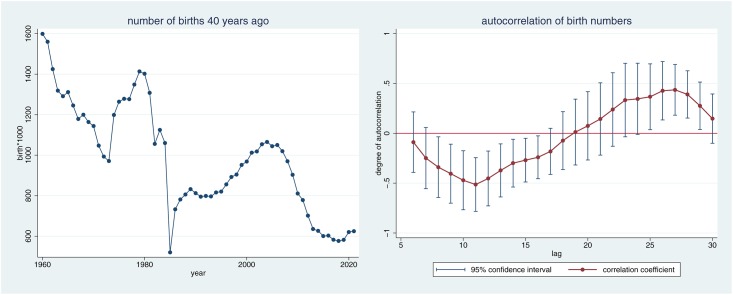
Development of birth cohorts over time.
